# Monte Carlo Dose Estimation of Absorbed Dose to the Hematopoietic Stem Cell Layer of the Bone Marrow Assuming Nonuniform Distribution Around the Vascular Endothelium of the Bone Marrow: Simulation and Analysis Study

**DOI:** 10.2196/68029

**Published:** 2025-07-16

**Authors:** Noriko Kobayashi

**Affiliations:** 1 Individual researcher, 4-16-18-1F Hamadayama Suginami-ku, Tokyo, 168-0065, Japan, 81 03-5929-7201

**Keywords:** stem cells, radiation, bone marrow, nuclides, noble gases

## Abstract

**Background:**

Recent studies have shown that hematopoietic stem cells (HSCs) are concentrated around the endothelium of the sinusoidal capillaries. However, the current dosimetry model proposed by the International Commission on Radiological Protection (ICRP) does not account for the heterogeneity of bone marrow tissue and stem cell distribution. If the location of the hematopoietic stem cell layer differs from previous assumptions, it is necessary to re-evaluate the dose. It is especially important for short-range alpha particles where the energy deposited in the target HSC layer can vary greatly depending on the distance from the source region.

**Objective:**

The objective of this study is to evaluate the red bone marrow doses assuming that the hematopoietic stem cell layer of the bone marrow is localized in the vascular endothelium.

**Methods:**

A model of the trabecular bone tissues in the cervical vertebrae was developed using the Particle and Heavy Ion Transport System code. Radiation transport simulations were performed for beta and alpha radionuclides as well as noble gases, and the absorbed doses to the stem cell layer within the perivascular HSC layer of the bone marrow from inhaled radionuclides were estimated. The estimated doses were then compared with the absorbed dose based on the ICRP 60 and ICRP 103 recommendations.

**Results:**

The absorbed doses to the bone marrow obtained from the model calculations were not significantly different from ICRP 60 and ICRP 103 for beta-nuclides. However, for alpha-nuclides, the absorbed doses were much lower than previously estimated. In addition, the contribution of red bone marrow and blood sources was greater than that of trabecular bone for alpha-nuclides. Noble gases in the red bone marrow may also affect the bone marrow stem cell layer.

**Conclusions:**

The bone marrow dose assessment for alpha nuclides and noble gases should be re-examined using a precise model based on computed tomography images from the perspective of occupational and public radiation protection.

## Introduction

Bone marrow is one of the most radiosensitive organs. Therefore, accurate dose assessment, considering bone microstructure and heterogeneous distribution of bone marrow tissues and cells, is critical. The International Commission on Radiological Protection (ICRP) model, currently adopted in Japan [1], assumes a homogeneous distribution of trabecular bone tissues and bone marrow stem cells.

Computational voxel phantoms have been introduced since the 2007 ICRP recommendation (ICRP 103) [2]. A precise skeletal model developed by Hough et al [3] using microcomputed tomography images of the trabecular spongiosa from an adult male cadaver has been incorporated into ICRP 133 [4]. However, hematopoietic stem cells (HSCs) are assumed to be uniformly distributed within the marrow cavities of hematopoietically active marrow [5].

Recent studies have shown that HSCs and immune cells are localized around the endothelium of bone marrow vessels [6]. One study reported that 85% of HSCs were located within 10 μm of bone marrow sinusoids in mice [7]. Kristensen et al [8] identified the microenvironment of HSCs and progenitors in the bone marrow by immunofluorescence staining of bone marrow tissue obtained from healthy volunteers. They found that the microenvironment of the HSCs is significantly enriched in sinusoids and megakaryocytes, while that of the progenitors is significantly enriched in capillaries, bone surfaces, and arteries.

Given this localized distribution of HSCs, it is necessary to re-evaluate the bone marrow dose, assuming that the HSC layer is localized around the sinusoidal capillaries of the bone marrow. This is especially important for short-range alpha particles, where the energy deposited in the target HSC layer can vary greatly depending on the distance from the source region.

Several bone marrow models have been developed for dosimetry of alpha-emitting radiopharmaceuticals, taking into account the microstructure of the bone marrow tissue. Hobbs et al [9] developed a simple geometric model of marrow cavities taking into account the distribution of bone marrow cells. They calculated the absorbed doses from ^223^Ra in the trabecular bone surface or in the endosteal layer (layer covering the surfaces of the trabecular bone) and found that the absorbed dose was predominantly deposited near the trabecular surface and “differed markedly from a standard absorbed fraction method.” Tranel et al [10] developed a cylindrical voxel bone marrow model with a blood vessel embedded in the center of the marrow and found that “the absorbed dose to the trabecular bone drops off quickly with increasing distance from the vessel wall, as the range of alphas ensures that the absorbed dose is minimal at distances greater than 100 μm.” However, both studies assume a homogeneous distribution of HSCs in the bone marrow cavity. Dosimetry that accounts for the arrangement of blood vessels in the bone marrow when the source is intravascular remains a challenge.

The aim of this paper is to evaluate the bone marrow dose when HSCs are localized around sinusoidal capillaries in the bone marrow and compare it with conventional values. A geometric model of trabecular bone and bone marrow tissue was constructed at the ㎛ scale, assuming that the HSC layer is located in the perivascular HSC layer of the sinusoids. The absorbed doses of the stem cell layer from blood and trabecular bone sources were then estimated for selected beta-nuclides, alpha-nuclides, and noble gases and compared with ICRP 60’s and ICRP 103’s specific absorbed fraction (SAF, fraction of radiation of energy emitted within the source region that is absorbed per mass in the target region) values. This is the first attempt at bone marrow dosimetry based on the assumption that the HSC layer is localized around sinusoidal capillaries in the bone marrow.

## Methods

### Geometric Modeling of Trabecular Bone and Bone Marrow Tissues

A model of the trabecular bone tissues in cervical vertebrae was created based on the data from JM-103 in the Japan Atomic Energy Agency (JAEA) Data/Code 2014‐017 [11], using the PHITS (Particle and Heavy Ion Transport System) code version 3.17 [12]. The JM-103 data were used because a detailed weight breakdown of bone tissue and blood was not available in the ICRP 89 [13]. The cervical vertebrae were selected for modeling because they are simple in shape and easy to model.

The height of the cervical vertebrae was estimated to be 9 cm based on the following assumptions: height 171 cm, length of the spine 52 cm (about 3/10 of the height), and cervical, thoracic, and lumbar vertebrae ratio of about 2:7:3. The weight of bone tissue and blood in the cervical spine was calculated by summing the values given in the JAEA Data/Code 2014‐017 [11]. Since the percentage of blood contained in each bone tissue was not reported, the amount of blood contained in the red bone marrow was calculated as 13.5% of the red bone marrow based on the percentages of the data reported in ICRP 89 [12] (7% of total blood for blood distributed in bone tissue and 4% for blood distributed in the red bone marrow) (Table 1). Data on the percentage of blood distributed in the sinusoids of the blood distributed in the red bone marrow were not available, so this was calculated at 89.4%, as shown in Table 2, using data from mouse bone marrow vessels by Bixel et al [14]. Material densities were set at 1.765 g/cm^3^ for trabecular bone [9] and 1 g/cm^3^ for red bone marrow, soft tissues, and blood.

**Table 1.** Weight of JM-103 cervical bone tissues.

Organ ID and name	Total body tissue^a^(g)	Cortical bone(g)	Trabecular bone (g)	Soft tissues (g)	Red bone marrow(g)	Blood (g)	Blood in red bone marrow^b^ (g)
140 Cervical vertebra_01	0.8	—^c^	0.2	0.6	0.5	0.1	0.1
141 Cervical vertebra_02	7.1	—	3.3	3.9	2.9	0.4	0.4
142 Cervical vertebra_03	40.7	13.7	8.6	18.3	13.7	2.2	1.8
143 Cervical vertebra_04	62.5	40	—	22.5	16.9	2.8	2.3
144 Cervical vertebra_05	47.5	36.1	—	11.4	8.5	1.6	1.2
145 Cervical vertebra_06	39.5	35.6	—	4	3	—	0.4
146 Cervical vertebra_07	8.6	8.6	—	—	—	—	—
Total	206.8	134.1	12.2	60.6	45.5	7.8	6.1

a Total body tissue = cortical bone + trabecular bone + soft tissues.

b Red bone marrow 1191.6 g, blood 281.2 g: 281.2 g × 4/7/1191.6 g = 13.5%.

c Not available.

**Table 2.** Percentage of blood distributed in the sinusoids calculated from bone marrow vessel data of mice.

Each structure and the geometrical conditions set for the calculation.	Vessel segments (n)	Mean diameter (μm)	Cross-sectional area of blood vessels ((b/2) ^2^ × 3.14) (μm^2^)^a^	Cross-sectional area of each blood vessels (c × a) (μm^2^)^b^	Percentage of total cross-sectional area(%)
Arterial vessel	9	8	50.2	452.2	3.7
Postarterial capillaries	5	7.8	47.8	238.8	2.0
Intermediate capillaries	6	11.2	98.5	590.8	4.9
Sinusoidal capillaries	31	21.1	349.5	10,834.2	89.4
Total	—^c^	—	—	12,116.0	—

a Calculation: (mean diameter/2)2 × 3.14.

b Calculation: cross-sectional area of blood vessels × number of segments.

c Not applicable.

Based on the statement of Saladine et al [15] that sinusoids are typically 30‐40 µm wide, the radius of the sinusoids was assumed to be 20 µm, and the number of vessels was assumed to be 40,000.

**Figure 1.**
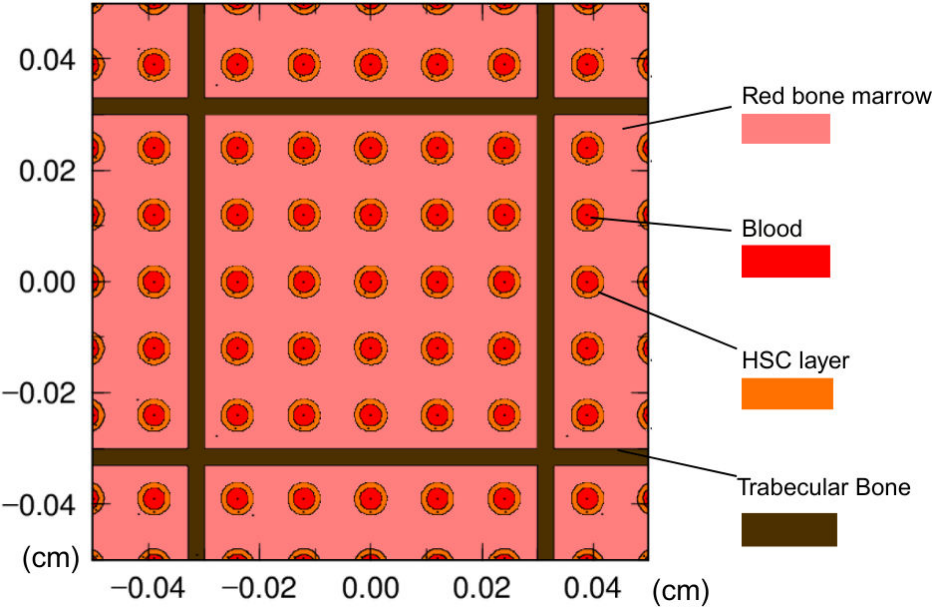
Geometry of the trabecular bone model constructed with the Particle and Heavy Ion Transport System code. HSC: hematopoietic stem cell.

The total number of lattices was set at 1600; the internal dimension of the lattice was set at 600 µm based on the data of Parfitt et al [16]; and the external dimension of the lattice was set to 630 µm based on the weight of the trabecular bone (Figure 1).

The target part of the organ was defined as the perivascular stem cell layer 10 µm from the vascular endothelium; Acar et al [7] reported that 85% of mouse HSCs were located within 10 µm of sinusoids, and Kunisaki et al [17] reported an average distance of 14.8 µm between the vascular endothelium and HSCs. The 10 µm from the surface of the trabecular bone was defined as the trabecular surface, and the inner 30 µm was defined as the trabecular volume. Since it is impossible to model the entire trabeculae, I modeled 9 grids of 25 vessels each, for a total of 225 vessels, and multiplied the value obtained from the PHITS calculation by a factor of 40,000/225.

### Radiation Transport Simulation and Absorbed Dose Calculation

^137^Cs, ^131^I, and ^90^Sr isotopes were selected for the calculation as beta-nuclides, ^223^Ra, ^239^Pu, ^238^U, ^232^Th, and ^222^Rn as alpha-nuclides, and ^133^Xe, ^135^Xe, and ^85^Kr as noble gases. Electron transport was simulated using PHITS code version 3.17 for β-radionuclides and noble gases, and alpha particles for alpha nuclides. The source regions were defined as blood, red bone marrow, trabecular bone volume, or trabecular bone based on the biokinetics of each radionuclide, and the target region was defined as the bone marrow stem cell layer 10 µm from the vascular endothelium.

For each radionuclide, electrons or alpha particles were generated in the source region, and the transferred energy distributed in the target region was calculated and converted to absorbed dose per incident particle (Gy/source). For the calculation of the beta nuclides, parameter e-type=28 was used for the source energy, which uses the DECDC [18] nuclear decay database (equivalent to ICRP 107 [19]) to obtain the energy spectra. The number of simulation trials was at least 10,000, and the statistical error in the target region was set to be less than 0.05. For the alpha radionuclide, the statistical error was set to be less than 0.5 due to the long computation time required when using the trabecular bone as a source. For ^232^Th, the calculation was stopped with the statistical error of 0.9 because the energy distributed from the trabecular bone sources to the perivascular area was very small, which will have only a limited effect on the results and discussion even though the statistical error is relatively large. The cut-off energy for photons and electrons was set at 5 keV. Bremsstrahlung, which is a type of X-radiation emitted by charged particles when they collide or near an atomic nucleus, was included in the simulation using the Electron Gamma Shower [20] mode.

### Calculation of the Number of Decays in Each Compartment

Assuming that 1 Bq (the International System of Units (SI) unit of radionuclide activity is the becquerel (Bq); 1 Bq=1 transformation/second) of radionuclide was inhaled, the number of decays in each compartment was calculated with R version 4.0.3 (R Foundation for Statistical Computing) [21] using the deSolve code [22] and the transfer coefficients presented in ICRP 56 [23], 67 [24], and 69 [25] for the current model, and those in ICRP 134 [26], 137 [27], and 141 [28] for the ICRP 103 model. The number of decays in each compartment of radionuclides transferred from the lungs to the blood was calculated for 15,800,000 minutes (10 years) for long-lived radionuclides and approximately 10,000 minutes for short-lived radionuclides. The choice of 10 years for long-lived nuclides instead of 50 years was made because of the limitations of the PC‘s performance (Intel Core i5-3337U CPU 1.8 GHz, with 7.90 GB of RAM), and 10,000 minutes for short-lived radionuclides.

For noble gases, the ICRP presents only a kinetic model for the radon dissolved in blood vessels and transported into the body. Since xenon and krypton are relatively easy to distribute in fat [29,30] as is radon [31], the transfer coefficients of radon were used for ^133^Xe, ^135^Xe, and ^85^Kr. Considering that the solubility of radon in water is twice that of xenon and 4 times that of krypton, it was assumed that 1/2 of the xenon and 1/4 of the krypton would be transferred to the blood.

The number of decays in red bone marrow blood was assumed to be proportional to the blood volume, which was 0.18% of the number of decays in whole body blood (red bone marrow blood volume in the cervical spine (volume of blood in red bone marrow of the cervical spine= 6.1g. volume of total blood in JM-103 model=3,410g. 6.1 g/3,410g=0.18%).

### Calculation of the Dose Absorbed in the Perivascular Stem Cell Layer of the Bone Marrow After Inhalation of Radionuclides

Assuming that 1 L of air was inhaled after 1 hour of exposure to air containing 1 Bq/m^3^ of radionuclides, the dose absorbed in the bone marrow perivascular stem cell layer was estimated by multiplying the absorbed dose determined in the section “Radiation Transport Simulation and Absorbed Dose Calculation” by the decay number calculated in the section “Calculation of the Number of Decays in Each Compartment.”

## Results

The absorbed doses calculated from the trabecular bone model and the comparison with the SAFs of ICRP 60 and ICRP 103 are shown in Multimedia Appendices 1-3. The absorbed dose to the perivascular HSC layer from each source was calculated for beta radionuclides (^137^Cs, ^131^I, and ^90^Sr) and compared with the doses estimated using the SAF and transfer coefficients in ICRP 60 and ICRP 103, presented in Multimedia Appendix 1.

The calculation results for alpha radionuclides (^223^Ra, ^239^Pu, ^238^U, ^232^Th, and ^222^Rn) are presented in Multimedia Appendix 2. For ^222^Rn, only results for the PHITS trabecular bone model are shown as SAFs for radon are not provided in ICRP 60 and ICRP 103.

Results for noble gases (^133^Xe, ^135^Xe, and ^85^Kr) are shown in Multimedia Appendix 3. As SAFs for noble gases are not provided in ICRP 60 and ICRP 103, only results for the PHITS trabecular bone model are shown. Table 3 summarizes the total absorbed doses to the perivascular HSC layer obtained from the PHITS calculation for each nuclide and the comparison with the ICRP 60 and ICRP 103 estimates.

**Table 3.** Summary of the calculated absorbed doses to the perivascular hematopoietic stem cell layer.

Nuclides		PHITS^a^ model (Gy/source)	ICRP 60(Gy/source)	ICRP 103(Gy/source)	ICRP 60/PHITS	ICRP 103/PHITS
Beta-nuclides						
^137^Cs		7.67E-09	6.83E-09	8.41E-09	0.9	1.1
^131^I		4.26E-12	1.28E-11	4.01E-12	1.8	0.9
^90^Sr		1.96E-08	3.43E-08	3.92E-08	1.4	2.0
Alpha-nuclides						
^223^Ra		1.88E-10	3.92E-09	8.19E-10	20.9	4.4
^239^Pu		1.32E-06	1.92E-05	3.23E-06	14.6	2.5
^238^U		4.45E-10	9.70E-08	1.03E-08	217.8	23.1
^232^Th		6.38E-07	2.26E-05	2.32E-06	35.4	3.6
^222^Rn		1.69E-11	—^b^	—	—	—
Noble gases						
^133^Xe		2.37E-13	—	—	—	—
^135^Xe		3.63E-13	—	—	—	—
^85^Kr		1.65E-13	—	—	—	—

a PHITS: Particle and Heavy Ion Transport System.

b Not available.

## Discussion

The results show that the absorbed doses to the bone marrow obtained from the model calculations were not significantly different from ICRP 60 and ICRP 103 for beta-nuclides. In contrast, for alpha-nuclides, the absorbed doses were much lower than previously estimated. For β-nuclides, the absorbed dose per decay was higher in the PHITS model for all 3 nuclides, but the absorbed dose was almost the same as in ICRP 60 because the number of decays in each compartment changed significantly due to changes in the biokinetic model and transfer coefficients.

**Figure 2.**
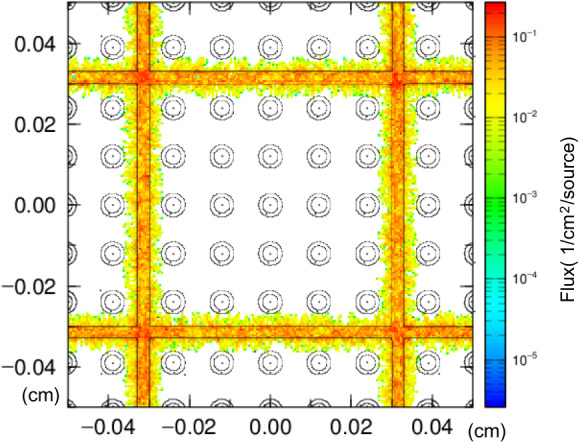
Particle and Heavy Ion Transport System simulation of ^239^Pu deposited on the trabecular bone surface to the perivascular hematopoietic stem cell layer.

For the alpha nuclides, few particles reached the perivascular HSC layer from the trabecular bone source due to their short range (Figure 2, nuclide: ^239^Pu, source organ: trabecular surface). This is consistent with the report by Tranel et al [10] that the range of alphas ensures absorbed dose is minimal at distances greater than 100 μm. Most of the dose to the perivascular HSC layer came from either the red bone marrow source or the blood source. Therefore, the dose calculated by the PHITS model was lower than the dose assessment based on the ICRP 60 recommendation, which assumes an absorbed fraction of 0.5 for the source trabecular surface and 0.05 for the trabecular bone volume. Compared to the dose estimated using the ICRP 103 SAF, the difference was smaller, about 2 to 23 times lower. Evaluation of alpha nuclides using a more accurate model is needed.

If HSCs are located in the perivascular HSC layer of the red bone marrow and are less susceptible to alpha radionuclides in bone sources, as suggested in Multimedia Appendix 2, the internal doses of alpha nuclides in epidemiological studies to date have been overestimated, and the actual doses to the red bone marrow may be lower. ^223^Ra has been used for the treatment of prostate cancer, and red bone marrow doses have been evaluated by Lassmann and Nosske [32], but actual doses may be lower. As with ^222^Rn, red bone marrow sources affect the stem cell layer. Radon biokinetics and bone marrow absorbed doses need to be evaluated, as reported by Sakoda et al [33].

Noble gases, for which internal exposure is not currently assessed, have lower absorbed doses per decay than beta and alpha nuclides, but exposure to large quantities may have radiation effects on the bone marrow stem cell layer. This may contribute to the radiation exposure of people living near accidents and nuclear power plants, where the effects of radiation exposure are controversial. A large amount of ^133^Xe was released into the environment during the Three Mile Island accident in 1979, but the exposure of nearby residents to xenon was assessed only for external exposure; internal exposure was not included in the radiation doses. Datesman [34] noted the discrepancy between the results of physical dosimetry and biodosimetry by cytogenetic analysis of residents living near the Three Mile Island nuclear power plant. Noble gases are 10 times more soluble in lipids than in nonlipid tissues [35], and Wang et al [36] reported that bone marrow fat accounts for about 10% of total fat in healthy adults. It has also been reported that bone marrow adipocytes are located adjacent to sinusoidal blood vessels and are hematopoietic [37]. The biokinetics of xenon and other noble gases in the body and the assessment of exposure to the bone marrow stem cell layer should be considered.

In terms of limitations, the trabecular bone model used in this paper is a simple model of part of the cervical vertebrae, although it is based on available human data. The model does not reflect differences in the mass of bone tissues according to location. The masses of bone tissues vary widely according to location in the bone, as shown in Table 4. The ratio of bone marrow and blood differs depending on the part of the bone, so the results obtained from the cervical vertebra model cannot be applied to the whole body. However, it is certainly necessary to perform a dose assessment that takes into account the fine structure of the bone and the location of the HSCs. A precise model based on microcomputed tomography images is required for dosimetry. In addition, since the transfer coefficients for noble gases are estimated from the coefficients for radon, it is necessary to construct a pharmacokinetic model based on actual measurements.

**Table 4.** Masses of bone tissues and blood of JM-103 by anatomical location.

Organ	Mass (g)						RBM/body tissue (%)	Blood/body tissue (%)
	Body tissue	RBM^a^	Cortical bone	Trabecular bone	Soft tissues	Blood		
Cranium	1346	91	774	308	264	28	6.8	2.0
Mandible	165	9	80	52	33	3	5.6	2.1
Cervical Vertebra	207	45	134	12	61	8	21.9	3.8
Thoracic Vertebra	654	187	315	77	262	32	28.7	4.9
Lumbar Vertebra	590	143	222	118	249	30	24.3	5.1
Sacrum	261	115	128	12	120	14	44.2	5.5
Clavicles	111	10	52	28	32	3	8.6	2.3
Scapulae	310	34	143	70	97	8	11.0	2.7
Sternum	107	36	39	21	47	6	33.9	5.2
Ribs	945	187	325	226	394	48	19.8	5.0
Os Coxae	1057	221	388	258	412	38	20.9	3.6
Humeri	589	29	282	122	193	10	4.9	1.7
Forearm	361	0	205	55	100	3	0.0	0.9
Wrist-Hand	220	0	115	36	69	2	0.0	1.0
Femora	1653	84	665	440	547	24	5.1	1.5
Tibiae-Fibulae-Patellae	1563	0	669	367	527	16	0.0	1.0
Ankle-Foot	872	0	299	262	313	9	0.0	1.0
Os Hyoideum	4	0	2	1	1	0	8.2	2.3
Total	11,014	1192	4837	2466	3721	281	10.8	2.6

a RBM: red bone marrow.

The bone marrow doses calculated with the PHITS trabecular bone marrow model, which assumes that the stem cell layer is located in the perivascular HSC layer of the sinusoids, showed that the absorbed doses from the bone marrow source and from the blood source were greater than those from trabecular bone sources for alpha nuclides. The total absorbed dose was lower than that estimated from the current ICRP models. The bone marrow dose assessments from internal exposure should be re-examined using a more detailed model of the trabecular bone marrow cavity, assuming heterogeneous distribution of HSCs and other bone marrow cells. It is also necessary to assess the effects of fat-soluble noble gases on HSCs in the bone marrow.

## Supplementary material

10.2196/68029 Multimedia Appendix 1 Absorbed doses to the perivascular hematopoietic stem cell layer for beta radionuclides calculated with the Particle and Heavy Ion Transport System model and comparison with doses estimated using specific absorbed fraction and transfer coefficients in ICRP 60 and ICRP 103.

10.2196/68029 Multimedia Appendix 2 Absorbed doses to the perivascular hematopoietic stem cell layer for alpha radionuclides calculated with the Particle and Heavy Ion Transport System model and comparison with doses estimated using specific absorbed fraction and transfer coefficients in ICRP 60 and ICRP 103.

10.2196/68029 Multimedia Appendix 3 Absorbed doses to the perivascular hematopoietic stem cell layer for noble gases calculated with the Particle and Heavy Ion Transport System model.

## Abbreviations

HSC: hematopoietic stem cell
ICRP: International Commission on Radiological Protection
JAEA: Japan Atomic Energy Agency
PHITS: Particle and Heavy Ion Transport System
SAF: specific absorbed fraction
