# Supplementary material for: Monte Carlo Dose Estimation of Absorbed Dose to the Hematopoietic Stem Cell Layer of the Bone Marrow Assuming Nonuniform Distribution Around the Vascular Endothelium of the Bone Marrow: Simulation and Analysis Study
Source: JMIRx Med. 2025 Jul 16;6:e68029. doi: 10.2196/68029 (PMC12286589; doi:10.2196/68029)
Supplement: Multimedia Appendix 1 [file xmed-v6-e68029-s001.docx]

Multimedia Appendix １

Absorbed doses to the perivascular HSC layer for beta readionuclides calculated with the PHITS model and comparison with doses estimated using SAF and transfer coefficients in ICRP60 and ICRP103

1. ^137^Cs

| ^137^Cs - PHITS model | | | | | |  |  |  |  |  |
| --- | --- | --- | --- | --- | --- | --- | --- | --- | --- | --- |
| a | b | c | d | e | f | | g | h | i |  |
| source | Number of decay  Type M | Mass in cervical vertebrae (g ) | Total mass in body  (g) | Number of decay in cervical vertebrae | Absorbed dose to perivascular HSC layer for 225 vessels  (Gy/source) | | Absorbed dose to perivascular HSC layer for 40000 vessels  (Gy/source) | Absorbed dose to perivascular HSC layer | Percentage of total absorbed dose  % |  |
| Plasma | 54,326 | 6 | 3,410 | 778 | 8.84E-11 | | 4.97E-13 | 3.87E-10 | 5% |  |
| Red blood cell | 381,394 |  |  |  |  |  |  |  |  |  |
| Red bone marrow | 407,957 | 45 | 1,192 | 15,401 | 7.72E-11 | | 4.34E-13 | 6.69E-09 | 87% |  |
| Trabecular bone surface | 406,381 | 12 | 2,466 | 1,978 | 5.32E-11 | | 2.99E-13 | 5.92E-10 | 8% |  |
| Total |  |  |  |  |  | |  | 7.67E-09 |  |  |

| **^137^Cs – ICRP60** | | | | | |
| --- | --- | --- | --- | --- | --- |
| j | k | l | m | n | o |
| source | Number of decay  Type F | Absorbed dose per  1 MeV | Absorbed dose per  0.243 MeV | Absorbed dose  (Gy/source) | Ratio of each organ (%) |
| Blood | 31,244 | 2.29E-15 | 5.56E-16 | 1.74E-11 | 0% |
| Body tissue A | 25,006 | 2.29E-15 | 5.56E-16 | 1.39E-11 | 0% |
| Body tissue B | 12,232,190 | 2.29E-15 | 5.56E-16 | 6.80E-09 | 100% |
| Total |  |  |  | 6.83E-09 | 100% |

| ^137^Cs – ICRP103 | | | | | | | |  |  |  |
| --- | --- | --- | --- | --- | --- | --- | --- | --- | --- | --- |
| p | q | r | | s | | t | | u | | v |
| Source | Number of decay  Type M | SAF to Red bone marrow for 0.2 MeV | Absorbed dose per  1MeV | | Absorbed dose  per 0.243MeV | | Absorbed dose  (Gy/source) | | Percentage of total absorbed dose  % | |
| Plasma | 54,326 | 2.81E-02 | 4.50E-15 | | 1.09E-15 | | 5.94E-11 | | 1% | |
| Red blood cell | 381,394 |  |  | |  | | 4.17E-10 | | 5% | |
| Red bone marrow | 407,957 | 3.72E-01 | 5.95E-14 | | 1.45E-14 | | 5.90E-09 | | 71% | |
| Trabecular bone surface | 406,381 | 1.29E-01 | 2.06E-14 | | 5.00E-15 | | 2.03E-09 | | 23% | |
| Total |  |  |  | |  | | 8.41E-09 | | 100% | |

a: Source compartment

b: Number of decays in each compartment, based on the transfer coefficients of ICRP134 and ICRP137

c: Weight of tissue in the cervical vertebrae (g)

d: Weight of tissue in the total body (g)

e: Number of decays in the cervical vertebrae (b×c/d)

f: Absorbed dose to the target per decay at the source, calculated for 225 blood vessels

g: Absorbed dose per decay for the entire cervical vertebrae (f×225/40000)

h: Absorbed dose to the target during the calculation period (g×e)

i: Percentage of h to total absorbed doses in perivascular HSC layer

j: Source compartment

k: Number of decays in each compartment based on ICRP60’s transfer coefficients

l: Absorbed fraction per MeV (1 MeV×1.60 × 10^-13^ (J MeV^−1^)×SAF, AF)

m: Absorbed dose per decay (l×mean energy)

n: Absorbed dose to the target during the calculation period (k×m)

o: Percentage of n to total absorbed doses

p: Source compartment

q: Number of decays in each compartment based on ICRP103 recommendation

r: SAFs provided in the SAF files of ICRP133

s: r× 1.6×10^-13^

t: Absorbed dose per decay (s×mean energy)

u: Absorbed dose to the target during the calculation period (q×t)

v: Percentage of u to total absorbed doses

1. ^131^I

| ^131^I - PHITS model | | | | | | | | |  |
| --- | --- | --- | --- | --- | --- | --- | --- | --- | --- |
| a | b | c | d | e | f | g | h | i |  |
| source | Number of decay  Type F | Mass in cervical vertebrae (g ) | Total mass in body  (g) | Number of decay in cervical vertebrae | Absorbed dose to perivascular HSC layer for 225 vessels  (Gy/source) | Absorbed dose to perivascular HSC layer for 40000 vessels  (Gy/source) | Absorbed dose in perivascular HSC layer | Percentage of total absorbed dose  % |  |
| Blood 1 | 2,780 | 6 | 3,410 | 5 | 9.33E-11 | 5.25E-13 | 2.65E-12 | 62% |  |
| Blood 2 | 1,684 | 6 | 3,346 | 3 | 9.33E-11 | 5.25E-13 | 1.61E-12 | 38% |  |
| Total |  |  |  |  |  |  | 4.26E-12 |  |  |

| ^131^I – ICRP60 | | | | | | |
| --- | --- | --- | --- | --- | --- | --- |
| j | k | | l | m | n | o |
| source | Number of decay  Type F | | Absorbed dose per  1 MeV | Absorbed dose per  0.1842 MeV | Absorbed dose  (Gy/source) | Percentage of total absorbed dose  % |
| Blood | 30,413 | 2.29E-15 | | 4.21E-16 | 1.28E-11 | 100% |
| Total |  | |  |  | 1.28E-11 |  |

|  | ^131^I – ICRP103 | | | | | | | |
| --- | --- | --- | --- | --- | --- | --- | --- | --- |
| p | | q | r | | s | t | u | v |
| Source | | Number of decay  Type F | SAF to Red bone marrow for 0.2 MeV | | Absorbed dose per  1MeV | Absorbed dose  per 0.2MeV | Absorbed dose  (Gy/source) | Percentage of total absorbed dose  % |
| Blood 1 | | 2,780 | 2.81E-02 | 4.50E-15 | | 8.99E-16 | 2.50E-12 | 62% |
| Blood 2 | | 1,684 | 2.81E-02 | 4.50E-15 | | 8.99E-16 | 1.51E-12 | 38% |
| Total | |  |  |  | |  | 4.01E-12 | 100% |

3. ^90^Sr

| ^90^Sr - PHITS model | | | | | | | | |  |  |
| --- | --- | --- | --- | --- | --- | --- | --- | --- | --- | --- |
| a | b | | c | d | e | f | g | h | i |  |
| source | | Number of decay  Type M | Mass in cervical vertebrae (g ) | Total mass in body  (g) | Number of decay in cervical vertebrae | Absorbed dose to perivascular HSC layer for 225 vessels  (Gy/source) | Absorbed dose to perivascular HSC layer for 40000 vessels  (Gy/source) | Absorbed dose to perivascular HSC layer | Percentage of total absorbed dose  % |  |
| Blood | 15,597 | | 6 | 3,410 | 28 | 9.28E-11 | 5.22E-13 | 1.48E-11 | 0% |  |
| Trabecular bone surface | 55,827 | | 12 | 2,466 | 272 | 5.29E-11 | 2.97E-13 | 8.08E-11 | 0% |  |
| Exch Trabecular bone volume | 1,481,146 | | 12 | 2,466 | 7,208 | 5.69E-11 | 3.20E-13 | 2.31E-09 | 12% |  |
| Nonexch Trabecular bone volume | 11,039,663 | | 12 | 2,466 | 53,721 | 5.69E-11 | 3.20E-13 | 1.72E-08 | 88% |  |
| Total |  | |  |  |  |  |  | 1.96E-08 |  |  |

| ^90^Sr – ICRP60 | | | | | | | | | | |
| --- | --- | --- | --- | --- | --- | --- | --- | --- | --- | --- |
| j | k | | l | m | | n | | o | | |
| source | Number of decay  Type F | | Absorbed dose per  1 MeV | Absorbed dose per  0.174 MeV | | Absorbed dose  (Gy/source) | | Percentage of total absorbed dose  % | | |
| Blood | 26,405 | | 2.27E-15 | 3.95E-16 | | 1.04E-11 | | 0% | | |
| Trabecular bone surface | 45,119 | | 8.00E-14 | 1.39E-14 | | 6.28E-10 | | 0% | | |
| Exch Trabecular bone volume | 602,246 | | 5.60E-14 | 9.74E-15 | | 5.87E-09 | | 17% | | |
| Nonexch Trabecular bone volume | 2,856,130 | | 5.60E-14 | 9.74E-15 | | 2.78E-08 | | 81% | | |
| Total |  |  | | |  | | 3.43E-08 | | 100% |  |

| ^90^Sr – ICRP103 | | | | | | |  |
| --- | --- | --- | --- | --- | --- | --- | --- |
| p | q | r | s | t | u | v | |
| Source | Number of decay  Type M | SAF to Red bone marrow for 0.2 MeV | Absorbed dose per  1MeV | Absorbed dose  per 0.174MeV | Absorbed dose  (Gy/source) | Percentage of total absorbed dose  % | |
| Blood | 15,597 | 2.81E-02 | 4.50E-15 | 7.82E-16 | 1.22E-11 | 0% | |
| Trabecular bone surface | 55,827 | 1.29E-01 | 1.06E-14 | 3.58E-15 | 2.00E-10 | 1% | |
| Exch Trabecular bone volume | 1,481,146 | 1.12E-01 | 1.79E-14 | 3.11E-15 | 4.61E-09 | 12% | |
| Nonexch Trabecular bone volume | 11,039,663 | 1.12E-01 | 1.79E-14 | 3.11E-15 | 3.44E-08 | 88% | |
| Total |  |  |  |  | 3.92E-08 | 100% | |
