# Supplementary material for: Monte Carlo Dose Estimation of Absorbed Dose to the Hematopoietic Stem Cell Layer of the Bone Marrow Assuming Nonuniform Distribution Around the Vascular Endothelium of the Bone Marrow: Simulation and Analysis Study
Source: JMIRx Med. 2025 Jul 16;6:e68029. doi: 10.2196/68029 (PMC12286589; doi:10.2196/68029)
Supplement: Multimedia Appendix 2 [file xmed-v6-e68029-s002.docx]

Multimedia appendix 2

Absorbed doses to the perivascular HSC layer for alpha radionuclides calculated with the PHITS model and comparison with doses estimated using SAF and transfer coefficients in ICRP60 and ICRP103

1. ^223^Ra

| ^223^Ra - PHITS model | | | | | | | | |  |
| --- | --- | --- | --- | --- | --- | --- | --- | --- | --- |
| a | b | c | d | e | f | g | h | i | |
| Source | Number of decay  Type M | Mass in cervical vertebrae (g ) | Total mass in body  (g) | Number of decay in cervical vertebrae | Absorbed dose to perivascular HSC layer for 225 vessels  (Gy/source) | Absorbed dose to perivascular HSC layer for 40000 vessels  (Gy/source) | Absorbed dose to perivascular HSC layer | Percentage of total absorbed dose  % | |
| Blood | 2,270 | 6 | 3,410 | 4 | 7.49E-09 | 4.21E-11 | 1.74E-10 | 93% | |
| Trabecular bone surface | 5,442 | 12 | 2,466 | 26 | 8.53E-11 | 4.80E-13 | 1.27E-11 | 7% | |
| Exch Trabecular bone volume | 7,551 | 12 | 2,466 | 37 | 4.02E-12 | 2.26E-14 | 8.32E-13 | 0% | |
| Nonexch Trabecular bone volume | 568 | 12 | 2,466 | 3 | 4.02E-12 | 2.26E-14 | 6.25E-14 | 0% | |
| Total |  |  |  |  |  |  | 1.88E-10 | 100% | |

| ^223^Ra – ICRP60 | | | | | |  |
| --- | --- | --- | --- | --- | --- | --- |
| j | k | l | m | n | o | |
| source | Number of decay  Type M | Absorbed dose per  1 MeV | Absorbed dose per  5.979 MeV | Absorbed dose  (Gy/source) | Percentage of total absorbed dose  % | |
| Blood | 538 | 2.27E-15 | 1.36E-14 | 7.31E-12 | 0% | |
| Trabecular bone surface | 7,176 | 8.00E-14 | 4.78E-13 | 3.43E-09 | 88% | |
| Exch Trabecular bone volume | 9,957 | 8.00E-15 | 4.78E-14 | 4.76E-10 | 12% | |
| Nonexch Trabecular bone volume | 748 | 8.00E-15 | 4.78E-14 | 3.58E-11 | 1% | |
| Total |  |  |  | 3.92E-09 | 100% | |

|  | ^223^ Ra – ICRP103 | | | | | | | | | | | | | | | |
| --- | --- | --- | --- | --- | --- | --- | --- | --- | --- | --- | --- | --- | --- | --- | --- | --- |
| p | q | | r | | s | | t | | | | u | | | | v |  |
| Source | Number of decay  Type M | | SAF to Red bone marrow for 6 MeV | | Absorbed dose per  1MeV | | Absorbed dose  per 5.979MeV | | | Absorbed dose  (Gy/source) | | | | Percentage of total absorbed dose | |  |
| Blood | 2,270 | | 2.87E-02 | | 4.59E-15 | | 2.74E-14 | | 6.23E-11 | | | 8% | | | |  |
| Trabecular bone surface | 5,442 | | 1.17E-01 | | 1.87E-14 | | 1.12E -13 | | 6.10E-10 | | | 74% | | | |  |
| Exch Trabecular bone volume | 7,551 | | 1.89E-02 | | 3.02E-15 | | 1.81E-14 | | 1.36E-10 | | | 17% | | | |  |
| Nonexch Trabecular bone volume | 568 | | 1.89E-02 | | 3.02E-15 | | 1.81E-14 | | 1.02E-11 | | | 1% | | | |  |
| Total |  |  | |  | |  | |  | | 8.19E-10 | | | 100% | | |  |

a: Source compartment

b: Number of decays in each compartment, based on the transfer coefficients of ICRP134 and ICRP137

c: Weight of tissue in the cervical vertebrae (g)

d: Weight of tissue in the total body (g)

e: Number of decays in the cervical vertebrae (b×c/d)

f: Absorbed dose to the target per decay at the source, calculated for 225 blood vessels

g: Absorbed dose per decay for the entire cervical vertebrae (f×225/40000)

h: Absorbed dose to the target during the calculation period (g×e)

i: Percentage of h to total absorbed doses in perivascular HSC layer

j: Source compartment

k: Number of decays in each compartment based on ICRP60’s transfer coefficients

l: Absorbed fraction per MeV (1 MeV×1.60 × 10^-13^ (J MeV^−1^)×SAF, AF)

m: Absorbed dose per decay (l×mean energy)

n: Absorbed dose to the target during the calculation period (k×m)

o: Percentage of n to total absorbed doses

p: Source compartment

q: Number of decays in each compartment based on ICRP103 recommendation

r: SAFs provided in the SAF files of ICRP133

s: r× 1.6×10^-13^

t: Absorbed dose per decay (s×mean energy)

u: Absorbed dose to the target during the calculation period (q×t)

v: Percentage of u to total absorbed doses

2. ^239^Pu

| ^239^Pu - PHITS model | | | | | | | | |  |
| --- | --- | --- | --- | --- | --- | --- | --- | --- | --- |
| a | b | c | d | e | f | g | H | i | |
| Source | Number of decay  Type M | Mass in cervical vertebrae (g ) | Total mass in body  (g) | Number of decay in cervical vertebrae | Absorbed dose to perivascular HSC layer for 225 vessels  (Gy/source) | Absorbed dose to perivascular HSC layer for 40000 vessels  (Gy/source) | Absorbed dose in perivascular HSC layer | Percentage of total absorbed dose  % | |
| Blood 0-2 | 243,885 | 6 | 3,410 | 444 | 7.09E-09 | 3.99E-11 | 1.77E-08 | 1% | |
| Trabecular bone surface | 29,168,202 | 12 | 2,466 | 141,938 | 1.32E-11 | 7.43E-14 | 1.05E-08 | 1% | |
| Trabecular bone volume | 6,328,741 | 12 | 2,466 | 30,797 | 1.02E-17 | 5.72E-20 | 1.76E-15 | 0% | |
| Trabecular bone marrow | 2,159,148 | 45 | 1,192 | 81,511 | 2.81E-09 | 1.58E-11 | 1.29E-06 | 98% | |
| Total |  |  |  |  |  |  | 1.32E-06 | 100% | |

| ^239^Pu – ICRP60 | | | | | |  |
| --- | --- | --- | --- | --- | --- | --- |
| j | k | l | m | n | o | |
| source | Number of decay  Type M | Absorbed dose per  1 MeV | Absorbed dose per  5.1 MeV | Absorbed dose  (Gy/source) | Percentage of total absorbed dose  % | |
| Blood | 195,809 | 2.29E-15 | 1.17E-14 | 2.28E-09 | 0% | |
| Trabecular bone surface | 39,800,554 | 8.00E-14 | 4.08E-13 | 1.62E-05 | 85% | |
| Trabecular bone volume | 9,980,277 | 8.00E-15 | 4.08E-14 | 4.07E-07 | 2% | |
| Trabecular bone marrow | 3,104,934 | 1.60E-13 | 8.16E-13 | 2.53E-06 | 13% | |
| Total |  |  |  | 1.92E-05 | 100% | |

|  | ^239^ Pu – ICRP103 | | | | | | |
| --- | --- | --- | --- | --- | --- | --- | --- |
| p | q | r | s | t | u | v |  |
| Source | Number of decay  Type M | SAF to Red bone marrow for  5 MeV | Absorbed dose per  1MeV | Absorbed dose  per 5.1MeV | Absorbed dose  (Gy/source) | Percentage of total absorbed dose  % | |
| Blood 0-2 | 243,885 | 2.87E-02 | 4.59E-15 | 2.34E-14 | 5.71E-09 | 0% | |
| Trabecular bone surface | 29,168,202 | 1.16E-01 | 1.86E-14 | 9.49E-14 | 2.77E-06 | 86% | |
| Trabecular bone volume | 6,328,741 | 1.44E-02 | 2.30E-15 | 1.17E-14 | 7.43E-08 | 2% | |
| Trabecular bone marrow | 2,159,148 | 2.17E-01 | 3.47E-14 | 1.77E-13 | 3.83E-07 | 12% | |
| Total |  |  |  |  | 3.23E-06 | 100% | |

3. ^238^U

|  | ^238^U - PHITS model | | | | | | | |
| --- | --- | --- | --- | --- | --- | --- | --- | --- |
| a | b | c | d | e | f | g | h | i |
| Source | Number of decay  Type M | Mass in cervical vertebrae (g ) | Total mass in body  (g) | Number of decay in cervical vertebrae | Absorbed dose to perivascular HSC layer for 225 vessels  (Gy/source) | Absorbed dose to perivascular HSC layer for 40000 vessels  (Gy/source) | Absorbed dose to perivascular HSC layer | Percentage of total absorbed dose  % |
| Blood | 4,252 | 6 | 3,410 | 8 | 5.65E-09 | 3.18E-11 | 2.54E-10 | 57% |
| Red blood cell | 3,423 | 6 | 3,410 | 6 | 5.65E-09 | 3.18E-11 | 1.91E-10 | 43% |
| Trabecular bone surface | 62,768 | 12 | 2,466 | 305 | 2.29E-17 | 1.29E-19 | 3.93E-17 | 0% |
| Trabecular bone volume | 596,632 | 12 | 2,466 | 2,903 | 9.45E-18 | 5.32E-20 | 1.54E-16 | 0% |
| Exch trabecular bone volume | 188,536 | 12 | 2,466 | 917 | 9.45E-18 | 5.32E-20 | 4.88E-17 | 0% |
| Total |  |  |  |  |  |  | 4.45E-10 | 100% |

| ^238^U – ICRP60 | | | | | | | | | |  |
| --- | --- | --- | --- | --- | --- | --- | --- | --- | --- | --- |
| j | k | l | | m | | n | | o |  |  |
| source | Number of decay  Type M | Absorbed dose per  1 MeV | Absorbed dose per  5.979 MeV | | Absorbed dose  (Gy/source) | | Percentage of total absorbed dose  % | | | |
| Blood | 4,353 | 2.27E-15 | 1.36E-14 | | 5.91E-11 | | 0% | | | |
| Red blood cell | 3,069 | 2.27E-15 | 1.36E-14 | | 4.17E-11 | | 0% | | | |
| Trabecular bone surface | 101,836 | 8.00E-14 | 4.78E-13 | | 4.87E-08 | | 50% | | | |
| Exch Trabeci;ar bpme volume | 302,474 | 8.00E-15 | 4.78E-14 | | 1.45E-08 | | 15% | | | |
| Nonexch Trabecular bone volume | 704,532 | 8.00E-15 | 4.78E-14 | | 3.37E-08 | | 35% | | | |
| Total |  |  |  | | 9.70E-08 | | 100% | | | |

|  | ^238^U – ICRP103 | | | | | |
| --- | --- | --- | --- | --- | --- | --- |
| p | q | r | s | t | u | v |
| Source | Number of decay  Type M | SAF to Red bone marrow for  4 MeV | Absorbed dose per  1MeV | Absorbed dose  per 4.1MeV | Absorbed dose  (Gy/source) | Percentage of total absorbed dose  % |
| Blood | 4,252 | 2.87E-02 | 4.59E-15 | 1.88E-14 | 8.00E-11 | 1% |
| Red blood cell | 3,423 | 2.87E-02 | 4.59E-15 | 1.88E-14 | 6.44E-11 | 1% |
| Trabecular bone surface | 62,768 | 1.16E-01 | 1.86E-14 | 7.63E-14 | 4.79E-09 | 47% |
| Trabecular bone volume | 596,632 | 1.04E-02 | 1.66E-15 | 6.82E-15 | 4.07E-09 | 40% |
| Exch Trabecular bone volume | 188,536 | 1.04E-02 | 1.66E-15 | 6.82E-15 | 1.29E-09 | 13% |
| Total |  |  |  |  | 1.03E-08 | 100％ |

4. ^232^Th

| ^232^Th - PHITS model | | | | | | | | |  |
| --- | --- | --- | --- | --- | --- | --- | --- | --- | --- |
| a | b | c | d | e | f | g | h | I | |
| Source | Number of decay  Type S | Mass in cervical vertebrae (g ) | Total mass in body  (g) | Number of decay in cervical vertebrae | Absorbed dose to perivascular HSC layer for 225 vessels  (Gy/source) | Absorbed dose to perivascular HSC layer for 40000 vessels  (Gy/source) | Absorbed dose to perivascular HSC layer | Percentage of total absorbed dose  % | |
| Blood | 28,759 | 6 | 3,410 | 52 | 5.30E-09 | 2.98E-11 | 1.55E-09 | 0% | |
| Trabecular bone surface | 23,970,084 | 12 | 2,466 | 116,643 | 2.14E-19 | 1.20E-21 | 4.98E-16 | 0% | |
| Trabecular bone volume | 66 | 12 | 2,466 | 0 | 2.62E-19 | 1.48E-21 | 0 | 0% | |
| Red bone marrow | 1,536,104 | 45 | 1,192 | 57,991 | 2.20E-09 | 1.10E-11 | 6.37E-07 | 100% | |
| Total |  |  |  |  |  |  | 6.38E-07 | 100% | |

| ^232^Th – ICRP60 | | | | | |  |
| --- | --- | --- | --- | --- | --- | --- |
| j | k | l | m | n | o | |
| source | Number of decay  Type M | Absorbed dose per  1 MeV | Absorbed dose per  4.1 MeV | Absorbed dose  (Gy/source) | Percentage of total absorbed dose  % | |
| Blood | 78,306 | 1.60E-13 | 6.56E-13 | 5.14E-08 | 0% | |
| Trabecular bone surface | 57,703,246 | 8.00E-14 | 3.28E-13 | 1.89E-05 | 84% | |
| Trabecular bone volume | 16,519,053 | 8.00E-15 | 3.28E-14 | 5.42E-07 | 2% | |
| Trabecular bone marrow | 4,662,328 | 1.60E-13 | 6.56E-13 | 3.06E-06 | 14% | |
| Total |  |  |  | 2.26E-05 | 100% | |

|  | ^232^ Th – ICRP103 | | | | | |  |
| --- | --- | --- | --- | --- | --- | --- | --- |
| p | q | r | s | t | u | v | |
| Source | Number of decay  Type S | SAF to Red bone marrow for  5 MeV | Absorbed dose per  1MeV | Absorbed dose  per 5.1MeV | Absorbed dose  (Gy/source) | Percentage of total absorbed dose  % | |
| Blood | 28,759 | 2.87E-02 | 4.59E-15 | 1.88E-14 | 5.41E-10 | 0% | |
| Trabecular bone surface | 23,970,084 | 1.16E-01 | 1.86E-14 | 7.61E-14 | 1.82E-06 | 79% | |
| Trabecular bone volume | 66 | 1.04E-02 | 1.66E-15 | 6.82E-15 | 4.50E-13 | 0% | |
| Red bone marrow | 1,536,104 | 4.91E-01 | 7.86E-14 | 3.22E-13 | 4.95E-07 | 21% | |
| Total |  |  |  |  | 2.32E-06 | 100% | |

5. 222Rn

| ^222^Rn - PHITS model | | | | | | | | |
| --- | --- | --- | --- | --- | --- | --- | --- | --- |
| a | b | c | d | e | f | g | h | i |
| Source | Number of decay | Mass in cervical vertebrae (g ) | Total mass in body  (g) | Number of decay in cervical vertebrae | Absorbed dose to perivascular HSC layer for 225 vessels  (Gy/source) | Absorbed dose to perivascular HSC layer for 40000 vessels  (Gy/source) | Absorbed dose to perivascular HSC layer | Percentage of total absorbed dose  % |
| Blood A | 4 | 6 | 3,410 | 0 | 7.40E-09 | 4.16E-11 | 0 | 0% |
| Blood V | 14 | 6 | 3,410 | 0 | 7.40E-09 | 4.16E-11 | 0 | 0% |
| Trabecular bone volume | 1 | 12 | 2,466 | 0 | 4.09E-13 | 2.30E-15 | 0 | 0% |
| Red bone marrow | 31 | 45 | 1,192 | 1 | 3.01E-09 | 1.69E-11 | 1.69E-11 | 100% |
| Total |  |  |  |  |  |  | 1.69E-11 | 100% |
