# Supplementary material for: Monte Carlo Dose Estimation of Absorbed Dose to the Hematopoietic Stem Cell Layer of the Bone Marrow Assuming Nonuniform Distribution Around the Vascular Endothelium of the Bone Marrow: Simulation and Analysis Study
Source: JMIRx Med. 2025 Jul 16;6:e68029. doi: 10.2196/68029 (PMC12286589; doi:10.2196/68029)
Supplement: Multimedia Appendix 3 [file xmed-v6-e68029-s003.docx]

Multimedia Appendix 3

Absorbed doses to the perivascular HSC layer for noble gasses calculated with the PHITS model

1. ^133^Xe

| ^133^Xe - PHITS model | | | | | | | | |
| --- | --- | --- | --- | --- | --- | --- | --- | --- |
| a | b | c | d | e | f | g | h | i |
| Source | Number of decay | Mass in cervical vertebrae (g ) | Total mass in body  (g) | Number of decay in cervical vertebrae | Absorbed dose to perivascular HSC layer for 225 vessels  (Gy/source) | Absorbed dose to perivascular HSC layer for 40,000 vessels  (Gy/source) | Absorbed dose to perivascular HSC layer | Percentage of total absorbed dose  % |
| Blood A | 2 | 6 | 3,410 | 0 | 1.05E-10 | 5.99E-13 | 2.18E-15 | 1% |
| Blood V | 7 | 6 | 3,410 | 0 | 1.05E-10 | 5.99E-13 | 7.49E-15 | 3% |
| Trabecular bone volume | 0 | 12 | 2,466 | 0 | 0 | 0 | 0 | 0% |
| Red bone marrow | 15 | 45 | 1,192 | 1 | 7.15E-11 | 4.02E-13 | 2.28E-13 | 96% |
| Total |  |  |  |  |  |  | 2.37E-13 | 100% |

a: Source compartment

b: Number of decays in each compartment, based on the transfer coefficients of ICRP134 and ICRP137

c: Weight of tissue in the cervical vertebrae (g)

d: Weight of tissue in the total body (g)

e: Number of decays in the cervical vertebrae (b×c/d)

f: Absorbed dose to the target per decay at the source, calculated for 225 blood vessels

g: Absorbed dose per decay for the entire cervical vertebrae (f×225/40000)

h: Absorbed dose to the target during the calculation period (g×e)

i: Percentage of h to total absorbed doses in perivascular HSC layer

2. ^135^Xe

| ^135^Xe - PHITS model | | | | | | | | |
| --- | --- | --- | --- | --- | --- | --- | --- | --- |
| a | b | c | d | e | f | g | h | i |
| Source | Number of decay | Mass in cervical vertebrae (g ) | Total mass in body  (g) | Number of decay in cervical vertebrae | Absorbed dose to perivascular HSC layer for 225 vessels  (Gy/source) | Absorbed dose to perivascular HSC layer for 40000 vessels  (Gy/source) | Absorbed dose to perivascular HSC layer | Percentage of total absorbed dose  % |
| Blood A | 2 | 6 | 3,410 | 0 | 1.19E-10 | 6.69E-13 | 2.44E-15 | 1% |
| Blood V | 7 | 6 | 3,410 | 0 | 1.19E-10 | 6.69E-13 | 8.37E-15 | 2% |
| Trabecular bone volume | 0 | 12 | 2,466 | 0 | 0 | 0 | 0 | 0% |
| Red bone marrow | 15 | 45 | 1,192 | 1 | 1.11E-11 | 6.22E-13 | 3.52E-13 | 97% |
| Total |  |  |  |  |  |  | 3.63E-13 | 100% |

3. ^85^Kr

| ^85^Kr - PHITS model | | | | | | | | |
| --- | --- | --- | --- | --- | --- | --- | --- | --- |
| a | b | c | d | e | f | g | h | i |
| Source | Number of decay | Mass in cervical vertebrae (g ) | Total mass in body  (g) | Number of decay in cervical vertebrae | Absorbed dose to perivascular HSC layer for 225 vessels  (Gy/source) | Absorbed dose to perivascular HSC layer for 40,000 vessels  (Gy/source) | Absorbed dose to perivascular HSC layer | Percentage of total absorbed dose  % |
| Blood A | 1 | 6 | 3,410 | 0.0 | 1.04E-10 | 5.87E-13 | 1.07E-15 | 1% |
| Blood V | 4 | 6 | 3,410 | 0.0 | 1.04E-10 | 5.87E-13 | 4.19E-15 | 3% |
| Trabecular bone volume | 0 | 12 | 2,466 | 0 | 0 | 0 | 0 | 0% |
| Red bone marrow | 8 | 45 | 1,192 | 0.3 | 9.41E-11 | 5.29E-13 | 1.60E-13 | 97% |
| Total |  |  |  |  |  |  | 1.65E-13 | 100% |
